# Absolute and Relative Training Load and Its Relation to Fatigue in Football

**DOI:** 10.3389/fpsyg.2017.00878

**Published:** 2017-06-06

**Authors:** Unai Zurutuza, Julen Castellano, Ibon Echeazarra, David Casamichana

**Affiliations:** ^1^Physical Education and Sport Department, Faculty of Education and Sport, University of the Basque CountryVitoria-Gasteiz, Spain; ^2^Physical Performance DepartmentBeasain, Spain; ^3^Faculty of Physiotherapy and Speech Therapy, Gimbernat-Cantabria University School Associated with the University of CantabriaTorrelavega, Spain

**Keywords:** team sports, training, physical load, physiological load, fatigue

## Abstract

The aim of the study was to assess the relationship of external and internal training load (TL) indicators with the objective and subjective fatigue experienced by 15 semi-professional football players, over eight complete weeks of the competition period in the 2015–2016 season, which covered microcycles from 34th to 41st. The maximum heart rate (HRmax) and maximum speed (Vmax) of all the players were previously measured in specific tests. The TL was monitored via questionnaires on rating of perceived exertion (RPE), pulsometers and GPS devices, registering the variables: total distance (TD), player load 2D (PL2D), TD at >80% of the V_max_ (TD80), TD in deceleration at < -2 m⋅sec-2 (TDD <-2), TD in acceleration >2 m⋅sec^-2^ (TDA >2), Edwards (ED), time spent at between 50 and 80% (50–80% HRmax), 80–90% (80–90% HRmax), and >90% of the HR_max_ (>90% HR_max_), and RPE both respiratory/thoracic (RPEres) and leg/muscular (RPEmus). All the variables were analyzed taking into account both the absolute values accumulated over the week and the normalized values in relation to individual mean competition values. Neuromuscular fatigue was measured objectively using the countermovement jump test and subjectively via the *Total Quality Recovery* (TQR) scale questionnaire. Analytical correlation techniques were later applied within the general linear model. There is a correlation between the fatigue experienced by the player, assessed objectively and subjectively, and the load accumulated over the week, this being assessed in absolute and relative terms. Specifically, the load relative to competition correlated with the physical variables TD (-0.279), PL2D (-0.272), TDD < -2 (-0.294), TDA >2 (-0.309), and sRPEmus (-0.287). The variables related to heart rate produced a higher correlation with TQR. There is a correlation between objectively and subjectively assessed fatigue and the accumulated TL of a player over the week, with a higher sensitivity being shown when compared to the values related to the demands of competition. Monitoring load and assessing fatigue, we are closer to knowing what the prescription of an adequate dose of training should be in order for a player to be as fresh as possible and in top condition for a match. Normalizing training demands with respect to competition could be an appropriate strategy for individualizing player TL.

## Introduction

The main aim of training is to provide a stimulus which will optimize the player/team’s performance during competition whilst minimizing the negative consequences of that training such as lack of freshness, fatigue, over-training, or injury (Gabbett et al., 2012). The load experienced by players in training and competition can provoke temporary metabolic, neuromuscular or mental fatigue (Campos and Toscano, 2014), reducing performance (Fessi et al., 2016) and increasing the possibility of injury to the player (Ehrmann et al., 2016). In fact, the inappropriate management of training loads (TDs) is emerging as one of the main risk factors in no contact injuries (Soligard et al., 2016).

However, appropriate doses of stimulus could improve performance and protect against possible injury (Gabbett et al., 2016). It is therefore vitally important for physical fitness and sports technicians to determine the optimum quantity of training required for the player to continue improving his/her fitness or to maintain it without putting at risk their freshness, and to reduce the probability of injury, with a view to the maximization of performance in competition.

By monitoring load (Akenhead et al., 2016), information can be obtained concerning the handling of its prescription to try to reduce, when appropriate, acute fatigue (thus improving freshness), so that performance does not decrease whilst avoiding placing the player at greater risk of injury (Gabbett, 2016). The search for an optimum relationship between load, fatigue-freshness and performance is no easy task, given that it concerns an individual process influenced by internal and external factors which are at times independent from the workload itself (Gabbett et al., 2012). In addition to knowing the external load placed on the players, it is necessary to discover how this affects each player (internal load) given that the same external load can have different repercussions in different players or even in the same player at different points in the season (Impellizzeri et al., 2005). Current scientific literature (Gaudino et al., 2013; Colby et al., 2014) presents different methods for controlling load levels (external and internal) in team sports players. That research used objective measurements such as GPS devices (Casamichana et al., 2013) or heart rate monitoring (HR) (Henderson et al., 2015), but also subjective measurements such as the rating of perceived exertion (RPE) (Los Arcos et al., 2014b).

As a consequence of imposed load, the performance of a player is temporarily reduced due to the fatigue which is generated. Fatigue is defined as any decrease in muscular performance associated with muscular activity (Nédélec et al., 2012a). There is currently (Gastin et al., 2013) widespread use of different methods (objective and/or subjective) to assess fatigue. To this effect, the procedure used repeatedly to objectively assess fatigue is a variety of vertical jumps, such as the countermovement jump test (CMJ) (McLean et al., 2010; Malone et al., 2015; Thorpe et al., 2015). Alternatively, subjective assessments of fatigue are done using questionnaires such as that of Hopper (Hooper and Mackinnon, 1995), variables associated to *Wellness* (Thorpe et al., 2016a), *Total Quality Recovery Scale* (TQR) (Kentta and Hassmen, 1998), *The Profile of Mood State* (McNair et al., 1971).

There is an increasing tendency to study (Thorpe et al., 2016b) the relationships between the TD borne by players and the fatigue which this produces, in an attempt to find the optimum load with which to increase physical fitness, allowing the soccer players to be fresh for the match whilst avoiding loads which by default or excess put him/her at risk of injury.

Accumulated load values are usually assessed in absolute terms (Gabbett and Ullah, 2012; Cross et al., 2016). To date, no research has normalized the TD to the mean values of the player in competition. This would allow the comparison of the TD demand placed on the player with the demands of competition, which have shown a high inter-individual variability (Schuth et al., 2016).

To that end, the aim of this research is to study the relationship between external and internal training load indicators (TL), in absolute values and relative to competition, with respect to the fatigue experienced by semi-professional football players measured using objective and subjective values. The results of this research could increase knowledge about how to manage the load imposed on players with a view to adjusting its prescription in order to optimize physical performance in competition.

## Materials and Methods

### Subjects

A total of 15 semi-professional male football players (Defenders = 5, Midfielders = 8 and Forwards = 2, goalkeeper did not take part in the study) took part in the study (age = 25.2 ± 3.0 years; height = 177.8 ± 5.6 cm; weight = 76.9 ± 6.5 kg) percentage of body fat (Möhr and Johnsen, 1972) was 11.6 ± 2.7% from group IV of the third division in the Spanish League. The players did, on average, 3–4 weekly training sessions and played one official match every weekend. The Ethics Committee of research with humans (CEISH) of the University of the Basque Country (UPV/EHU) gave its institutional approval of the study. In accordance with the protocol, before taking part in the study, all the players involved signed an informed consent form. Both the participants and the team’s technical body were kept informed at all times about the procedure and possible risks and benefits of the study.

### Training Sessions and Competition Matches

All the training sessions and competition matches were monitored during the microcycles of the study. In total, 250 recordings were made in 20 training sessions (16.7 ± 3.6 per player) and 72 recordings from eight matches (4.9 ± 2.1 per player). The individual session or match recordings were grouped into microcycles, with a total of 69 weekly recordings (4.6 ± 1.3 per player). In order to calculate the mean value of the demands in competition, all the values were normalized to a 90 min match (mean match duration recorded per player ±SD).

### Heart Rate

In all the training sessions and competition matches heart rate (HR) was recorded via a short range telemetry system (Polar Team2 Pro System, Polar Electro Oy, Kempele, Finland). The reliability of the devices used in this study has been reported in previous studies (Macleod and Sunderland, 2012). To quantify the internal load from the HR the Edwards (1993) method was used. The Edwards method distributes the exertion of the HR in five different zones. Each zone has an established value (50–60% HR_max_ = 1, 60–70% HR_max_ = 2, 70–80% HR_max_ = 3, 80–90% HR_max_ = 4, 90–100% HR_max_ = 5) which are later added together.

To calculate the maximum HR for each player, a maximal progressive test was carried out on a treadmill with a HR monitor, beginning with a speed of 8 km/h^-1^ which was increased at a rate of 1 km/h^-1^ every minute until the point of physical exhaustion was reached (Graff, 2002). Furthermore, the minutes spent in each zone were taken into account in the following intensity ranges (Henderson et al., 2015): time spent between 50 and 80% of the maximum HR (50–80% HRmax), time spent between 80 and 90% of the maximum HR (80–90% HRmax); and time spent at more than 90% of the maximum HR (>90% HRmax).

### Perceived Exertion Response

Once the training and/or match was finished, the players had to complete a subjective RPE. The RPE questionnaire used was a translation into Spanish of the Borg scale of 0–10 points modified by Foster (Foster et al., 2001), adapted to distinguish between perceived respiratory/thoracic exertion (RPEres) and the perceived exertion in legs/muscular (RPEmus) (Los Arcos et al., 2014b).

The players were able to respond with a plus symbol (for example 5+ means 5.5) next to the unit of assessment. The assessment was carried out 10 min after the end of the session or match (Ngo et al., 2011). Afterward, the value obtained in each of the scales was multiplied by the duration of the session or match (including warm-up and rests or pauses, but excluding cool down) to obtain the following variables (sRPEres and sRPEmus).

### Physical Variables

The players’ external load was monitored using GPS devices (Minimax S4, Catapult Innovations, Docklands, VIC, Australia, 2010) which function at a sampling frequency of 10 Hz and contain a 100 Hz triaxial accelerometer. The reliability and validity of the devices used in this study have been reported in previous studies (Castellano et al., 2011b; Gale-Ansodi et al., 2016). The mean (±SD) number of satellites during data collection was 12.5 (±0.6). The device was attached to the upper back of each player using a special harness. The GPS devices were activated 15 min before the start of each session or match, in accordance with the manufacturer’s instructions. The data from the GPS devices was later downloaded to a PC to be analyzed using the Sprint v5.1.4 software package (Catapult Innovations, Docklands, VIC, Australia, 2010).

The following physical variables were studied: (a) TD, total distance (TD) in m; (b) TD80, distance covered at more than 80% of maximum speed (V_max_) in m; (c) PL2D, player load 2D [in arbitrary units (AUs)]; (d) TD80%, percentage of distance covered at more than 80% of V_max_ (in %); (e) TDD < -2, TD in deceleration under -2 m/sec^-2^ (in m); and (f) TDA >2, TD in acceleration over 2 m/sec^-2^ (in m).

### Assessment of Neuromuscular Fatigue

To assess neuromuscular fatigue, as in previous work (McLean et al., 2010), a test was carried out (vertical bipedal jump with countermovement and with hands on hips) using the previously validated app *My Jump v.1* (Balsalobre-Fernández et al., 2015). The protocol followed is similar to that of Malone et al. (2015). Prior to the test the players did a standard warm up including a 5 min low speed run with dynamic exercises and two 20 m progressions followed by three repeats of the jump.

The best value obtained in the 14 tests of the trial was used to calculate the maximum CMJ (CMJ_max_) of each player. Furthermore, in each microcycle the level of absolute fatigue (FATabs) of each player was calculated by discovering the percentage of the value obtained by the player on the pre-match session day (always carried out 24 h before the next match) with respect to the CMJ_max_ value. The following formula was used to calculate the absolute fatigue value: CMJ_pre_/CMJ_max_. To calculate relative fatigue (FATrel) the formula CMJ_pre_/CMJ_post_ was used. This second fatigue value was calculated in order to know specifically whether the load accumulated in the week prior to the one studied had any repercussion on the freshness or objective fatigue of the player. The coefficient of variation (CV) for each of the CMJ tests was of between 0.0 and 7.7%.

### Assessment of Subjective Fatigue

The subjective questionnaire TQR scale (Kentta and Hassmen, 1998) was used as a subjective measurement to assess the fatigue suffered by the players. The questionnaire was given to the players 10 min before the start of training or pre-match warm-up. The players had to complete the TQR by answering the question “how recovered do you feel?” on a scale of 0–10, with 0 being rested and 10 extremely good recovery.

### Procedure

This observational study was carried out during the competitive phase (March–April) of the 2015–2016 season during the microcycles from 34 to 41°. All training and matches were monitored via pulsometers and GPS. Two of the microcycles were excluded from the analysis as not all of the sessions were present. Before beginning the trial, the players underwent a maximal progressive resistance test on a treadmill (in laboratory) to calculate the maximum HR of each player and a 40 m speed test on the training ground whilst wearing the GPS devices. Furthermore, where higher values in peak speeds were detected, these were taken as the V_max_ of the player.

During the study period, performance in a CMJ test was recorded both on the first training day of the week (with a minimum of 48 h with respect to competition) and the last (24 h prior to the next competition). The test was always carried out indoors and the players previously familiarized with it. It was decided not to include the CMJ test for matches for two reasons – one was the lack of adequate facilities in away matches and the second was due to the difficulty in getting the players to carry out maximal tests on competition days, despite these having a low impact on fatigue. Furthermore, before beginning the first or the last training session of the week or pre-match warm-up, the players completed the TQR questionnaire (TQRpost, TQRpre, and TQRcomp, respectively). Finally, after the training session or match they completed the RPE questionnaire.

For the correlation analysis between load and fatigue, both the absolute values accumulated through the week and the accumulated values normalized to individual competition were used. The competitive reference values were obtained from the competitions recorded during the same trial. For this, the mean values of each player in competition were used as reference.

### Statistical Analysis

Starting from the relative values of the different physical, physiological and perception of exertion variables, correlation analysis techniques were implemented within the general linear model. The results are shown as mean and standard deviation (±SD). The Pearson correlation coefficient was calculated to determine whether there was a relationship, and if this was significant among the analyzed variables. To interpret the results, threshold values for the Pearson correlation coefficient used by Salaj and Marckovic (2011) were used: low (*r* ≤ 0.3), moderate (0.3 < *r* ≤ 0.7) to high (*r* >0.7). The statistical analysis was conducting using SPSS v.23 (IBM, Corp., Chicago, IL, United States). Significance level was fixed at 0.05 (*p* < 0.05).

## Results

**Table 1** shows the mean and ±SD values for each of the variables obtained by the players in the matches played during the trial. It also shows the mean and (±SD) values of the load accumulated by microcycle, normalized to the demand of competition. It can be seen that in all the analyzed variables, except that of TD80, the accumulated weekly load was higher than the mean load in competition (a value of 100% means that the demands of competition are repeated for this variable).

**Table 1:** Mean and standard deviation values (±SD) for the profile of the external and internal demand on the players in competition and of the weekly training load (TD) in percentage values with respect to the individual demands of competition.

Variables (units)	Competition demand (units)		Weekly training demand (%)	
	Mean	±*SD*	Mean	±*SD*
TD (m)	9061.5	935.5	151.4%	40.4%
TD80 (m)	99.9	66.7	48.7%	39.6%
PL2D (AU)	534.7	83.2	177.7%	45.6%
TDD < -2 (m)	220.3	49.5	145.5%	43.8%
TDA >2 (m)	303.6	66.1	157.7%	42.3%
ED (AU)	345.4	31.5	144.5%	46.3%
>90% HRmax (min)	17.7	13.3	112.2%	122.6%
80–90% HRmax (min)	39.6	8.8	102.6%	38.6%
50–80% HRmax (min)	25.4	13.9	693.8%	478.0%
RPEres (AU)	6.5	1.2	177.9%	56.4%
RPEmus (AU)	6.8	1.3	167.2%	46.4%
sRPEres (AU)	581.9	103.9	165.1%	59.6%
sRPEmus (AU)	611.3	114.7	158.3%	48.3%

*TD is total distance, PL2D is two dimension player load, TD80 is TD at more than 80% of the maximum speed (V_*max*_), TDD < -2 is TD in deceleration below -2 m/sec^*-2*^, TDA >2 is TD in acceleration above 2 m/sec^*-2*^, ED is Edwards, 50–80% HRmax is the time spent between at 50–80% of the HR_*max*_, 80–90% HRmax is the time spent between 80 and 90% of the HR_*max*_, >90% HRmax is the time spent at more than 90% of the HR_*max*_, RPEres is the perceived exertion response (respiratory/thoracic), RPEmus is the perceived exertion response (leg/muscular), sRPEres is the perceived exertion response (respiratory/thoracic) multiplied by the minutes in the session, and sRPEmus is the perceived exertion response (leg/muscular) multiplied by the minutes in the session.*

**Table 2** shows the values obtained in the different CMJ tests carried out during the trial together with assessment of neuromuscular fatigue (FATabs and FATrel) and the subjective assessments of the state of fatigue (TQRpost, TQRpre, and TQRcomp). As it can see when TQR scale are closed to the matches the values of subjective fatigue are higher, that is, player finished the week with better wellness.

**Table 2:** Mean and standard deviation values (±SD) of the objective and subjective fatigue variables.

	CMJpost (cm)	CMJpre (cm)	CMJmax (cm)	FATabs (%)	FATrel (%)	TQRpost (AU)	TQRpre (AU)	TQRcomp (AU)
Mean	34.8	34.7	37.4	0.93	0.99	7.34	7.44	7.91
±SD	3.6	3.6	3.4	0.04	0.05	1.80	1.26	1.25

*CMJpost is countermovement jump carried out post-competition, CMJpre is countermovement jump carried out pre-competition, CMJmax is the maximum value of countermovement jump obtained in the trial, FATabs is absolute fatigue, FATrel is relative fatigue, TQRpost is the TQR questionnaire completed on the first day of training, TQRpre on the day before competition and TQRcomp done on match day.*

**Table 3** shows the correlations between objective fatigue with the 13 load variables studied, both in absolute values and relative to the competitive demand for each player. The values show that the objectively measured fatigue (FATabs and FATrel) correlated only with some of the internal and external load variables, when the load was assessed in absolute and relative terms. Among the physical variables, only TDA >2 is moderately correlated with the FATabs variable, while the other variables that are significantly correlated with FATabs present low correlations. There were higher correlations between relative values of the match demands than when were used absolute values. Subjective assessment of fatigue (TQRpre and TQRcomp) obtained correlations in a moderate range and were significantly high (*p* < 0.01) for the three HR variables.

**Table 3:** Values of the Pearson correlations of the objective absolute (FATabs), relative (FATrel) and subjective fatigue variables on the day before (TQRpre) and on competition day (TQRcomp) in relation to the accumulated weekly values of the absolute load variables and those relative to competition.

Variables	FATabs		FATrel		TQRpre		TQRcomp	
	Rel	Abs	Rel	Abs	Rel	Abs	Rel	Abs
TD	–0.279ˆ*	–0.234	–0.233	–0.186	–0.046	–0.079	–0.190	–0.199
PL2D	–0.272ˆ*	–0.153	–0.192	–0.095	0.000	0.233	–0.144	0.015
TD80	–0.178	–0.221	–0.161	–0.277ˆ*	–0.034	0.016	0.095	0.055
TDD < -2	–0.294ˆ*	–0.278ˆ*	–0.251	–0.241	–0.029	–0.089	–0.135	–0.234
TDA >2	–0.309ˆ*	–0.283ˆ*	–0.227	–0.234	–0.036	–0.154	–0.134	–0.250
ED	–0.170	–0.138	–0.123	–0.129	0.195	0.215	0.075	0.109
>90% HRmax	0.074	0.042	0.079	–0.067	0.233	0.436ˆ**	0.193	0.288ˆ*
80–90% HRmax	0.031	–0.072	0.007	–0.033	0.350ˆ**	0.081	0.337ˆ*	0.164
50–80% HRmax	0.061	–0.302ˆ*	–0.090	–0.219	0.114	–0.186	0.146	–0.268ˆ*
RPEres	–0.039	–0.085	–0.045	0.096	0.022	–0.039	0.051	0.101
RPEmus	–0.186	–0.176	–0.086	–0.057	–0.021	–0.164	–0.052	–0.163
sRPEres	–0.166	–0.214	–0.141	–0.012	–0.037	–0.096	–0.032	0.021
sRPEmus	–0.287ˆ*	–0.283ˆ*	–0.174	–0.146	–0.086	–0.215	–0.120	–0.226

*TD is total distance, PL2D is player load 2D, TD80 is TD at more than 80% of maximum speed (V_*max*_), TDD < -2 is TD in deceleration below -2 m/sec^*-2*^, TDA >2 is TD in acceleration above 2 m/sec^*-2*^, ED is Edwards, >90% HRmax is the time spent at more than 90% of the HR_*max*_, 80–90% HRmax is the time spent between 80 and 90% of the HR_*max*_, 50–80% HRmax is the time spent between 50 and 80% of the HR_*max*_, RPEres is the perceived exertion response (respiratory/thoracic), RPEmus is the perceived exertion response (leg/muscular), sRPEres is the perceived exertion response (respiratory/thoracic) multiplied by the minutes in the session, and sRPEmus is the perceived exertion response (leg/muscular) multiplied by the minutes in the session. ^∗^*p* < 0.05 and ^∗∗^*p* < 0.01 (bilateral).*

## Discussion

The aim of this research was to study the relationship between the TD, from external and internal indicators, and the objective and subjective assessment of fatigue in semi-professional football players. This is the first piece of work which relates the load placed on semi-professional footballers in terms relative to the demands of competition with the fatigue accumulated in each microcycle of the competitive period, calculated using an objective CMJ test and also via the subjective perception of recovery quality (TQR).

The main results of the study can be summarized as follows: (1) normalizing training demands with respect to competition could be an appropriate strategy for individualizing player TD, (2) both the use of objective (from CMJ) and subjective (from TQR) fatigue indicators proved to be related to the load borne by players in the weekly microcycle.

The application of this procedure of individually monitoring TD and fatigue in players can be applied to load adjustment for each of the variables. The aim would be, on the one hand, to avoid players being fatigued on match day, and on the other hand, to increase the status of training among players, optimizing physical fitness and thus being able to give maximum performance on competition day. Previous studies (Impellizzeri et al., 2005; McLean et al., 2010; Nédélec et al., 2012b; Casamichana et al., 2013; Gastin et al., 2013; Gaudino et al., 2013; Colby et al., 2014; Los Arcos et al., 2014a; Henderson et al., 2015; Malone et al., 2015; Thorpe et al., 2015; Akenhead et al., 2016; Gabbett, 2016; Gabbett et al., 2016) have analyzed the load placed on players in training and matches, but none have compared the load accumulated by the players in a training microcycle normalized to the physical demands of competition, despite being a practice used by elite teams as a means of *training status* (Akenhead and Nassis, 2016).

The decision to take the physical demands of competition as an individual reference is due to the probability that similar doses of training (absolute load) do not suppose the same percentage in relation to that which competition demands of each player (% of the match). This is not only because of differences in imposed demands on the players depending on their position on the pitch (Di Salvo et al., 2007), but also due to inter-individual variations (Impellizzeri et al., 2005; Castellano and Blanco-Villaseñor, 2015) even among those playing in the same position. To consider only the demands of training in absolute values could lead to inappropriate decisions being taken in the prescription of TD, over-stimulating certain variables in some players whilst other players may not be sufficiently stimulated in relation to the values of some variables in competition.

This gives rise to a new hypothesis regarding the need to individualize the variables which can affect a player’s fatigue or recovery, which will require further research. It is known that each player assimilates loads in a different way, due to his/her past and present characteristics (Impellizzeri et al., 2005), which provokes a particular state of fatigue which could be conditioned by the type of demand variable (e.g., those related to speed, acceleration/deceleration or metabolic system). That is why it is essential to individualize training as far as possible in order to strengthen collective training and thus optimize competition performance.

In order to normalize the weekly load, in this work it was decided to take as a reference the mean values of each player in competition, whilst being aware that these competition demands present a moderate-elevated variability (Castellano and Blanco-Villaseñor, 2015) in response to numerous situational variables such as place, current score or the quality of the teams (Castellano et al., 2011a). All of these could provoke a demand on the players at specific times which is higher than the estimated match average.

However, the quantity of load placed on the players should be conditioned by what they are able to assimilate, in order not to avoid over-training or increasing the probability of injury (Gabbett, 2016). To avoid unwanted negative effects from the load, it is necessary to study the player’s accumulated fatigue. Special attention is currently being paid (McLean et al., 2010; Gabbett and Ullah, 2012) to the assessment of neuromuscular fatigue via a simple and objective vertical jump test (i.e., CMJ).

Assessment of neuromuscular fatigue from CMJ may not be sensitive when the aim is to compare acute fatigue from a football training session (difference in a test of jump over height reached in pre with respect to post-training) (Malone et al., 2015) perhaps because football training usually involves multidimensional demands (Gaudino et al., 2015). In our research, the neuromuscular fatigue measured with CMJ wassensitive to the different percentages of load borne by the players during the training week.

Along the same lines, Gathercole et al. (2015) in his study found significant correlations between different microcycles for the CMJ variable which measures neuromuscular fatigue. Although more research is needed, assessment of neuromuscular fatigue via CMJ, or other tests, could be a useful tool for adjusting optimum TD, by which the technical team could ensure that their players are fresh when they come to compete.

This innovative study has also analyzed variables connected to accelerations and decelerations in which correlations with fatigue have also been found. To be more specific, the variables related to the neuromuscular dimension (PL and accelerations and decelerations) showed a greater sensitivity (correlation) with this objective jump test.

The use of questionnaires such as the TQR has allowed us to discover the player’s degree of subjective recovery at the end of the microcycle, which provides information on the fatigue generated in the player during the week. Despite there being practically no differences in the TQRpost with respect to the end of the week (TQRpre), it is worth pointing out that in the variables related to HR, and therefore the cardiovascular energy system, it was the TQR questionnaire which showed a higher sensitivity to the changes.

This suggests that it may be interesting to consider that just as different dimensions of TD are monitored, it could be relevant to have various tools available with which to assess the state of player fatigue or recovery, which would deal with different dimensions of the fatigue generated.

One of the limitations of the study was the relatively low number of recordings of training session load and match load. A higher number of recordings would have provided more information about each player and therefore established particular load-fatigue relationships for each one, in the search for the adequate dose and typology of TD for each player.

We should also highlight that using a higher number of players in the study, apart from providing information about their physical condition, would have shown how far players with different ability or fitness present a particular load-recovery relationship. This would thus allow attention to be paid to the capacity for bearing load and/or being fresher in competition or having better recovery after the match (Rabbani and Buchheit, 2016).

Finally, it should also be underlined that the reduced sample group did not allow the incorporation of variables with which to differentiate the players who played a match in the week prior to the studied microcycle. It is probable that different states of recovery at the beginning of the week could have conditioned some of the results of this work.

### Practical Applications

With the information obtained from the monitoring of TD and assessment of fatigue (objective and subjective), we are closer to knowing what the prescription of an adequate dose of training should be in order for a player to be as fresh as possible and in top condition for a match.

## Conclusion

The main conclusion of this study is that in those microcycles where the players accumulated a greater TD or high values in the load indicators normalized to those demanded in competition, the players showed a higher level of neuromuscular fatigue, measured with CMJ. However, the players were able to recover practically the same CMJ values (measured with the FATrel) as at the beginning of the week prior to competition. This research provides a better understanding of the load-fatigue relationship with respect to competition demands. Information about external objective load (distance, speed, and acceleration/deceleration), internal objective load (HR) and internal subjective load (RPE) on the one hand, and the objective (CMJ) and subjective (TQR) indicators for fatigue assessment on the other, can help trainers to better understand and adequately manage training status and player freshness throughout the training process. Finally, it would be necessary to know whether the load borne by the players in the weekly training process maintains or improves their fitness, and thus discover whether management of the load-fatigue binomial produces an improvement in the players’ physical performance.

## Author Contributions

UZ: design, data getting, analysis, redaction; JC: design, analysis, redaction; IE: data getting, redaction; DC: design, redaction.

## Conflict of Interest Statement

The authors declare that the research was conducted in the absence of any commercial or financial relationships that could be construed as a potential conflict of interest.
